# Systems Biology and Cancer Prevention: All Options on the Table

**DOI:** 10.4137/grsb.s1114

**Published:** 2008-10-10

**Authors:** Simon Rosenfeld, Izet Kapetanovic

**Affiliations:** DHHS/NIH/National Cancer Institute/Division of Cancer Prevention, EPN, 6130 Executive Blvd, Bethesda, MD 20892, U.S.A

**Keywords:** cancer prevention, systems biology, mathematical modeling, biological networks, genetic regulation, general systems theory, dynamical stability

## Abstract

In this paper, we outline the status quo and approaches to further development of the systems biology concepts with focus on applications in cancer prevention science. We discuss the biological aspects of cancer research that are of primary importance in cancer prevention, motivations for their mathematical modeling and some recent advances in computational oncology. We also make an attempt to outline in big conceptual terms the contours of future work aimed at creation of large-scale computational and informational infrastructure for using as a routine tool in cancer prevention science and decision making.

## Introduction

Cancer is a collective term for a number of multi-factorial and heterogeneous diseases characterized by uncontrolled cellular growth. In the multi-step process, normal cells are initiated and transition through hyperplasia, different degrees of dysplasia and carcinoma *in situ* and eventually become invasive to adjacent tissue and metastasize to other organs and tissues. Cancer prevention aims to disrupt oncogenesis by chemical, biological, or nutritional intervention and thereby prevent, reverse or delay the development or recurrence of cancer. Primary prevention aims to block initiation and the secondary prevention strives to delay or reverse promotion or progression of carcinogenesis.

Biological systems (organism, organ, tissue, cellular, subcellular, molecular systems) are comprised of multiple interactive complex networks with redundant, convergent and divergent signaling pathways including numerous positive and negative feedback loops. They may be represented by abstract biological networks which aim to depict the essential elements and activities of the former via integrative and dynamic simulations. Systems biology represents an integrated approach to understand functions of biological systems and effects of perturbations on them.

Attempts to inject systemic views into biology have a long history. For example, an interesting perspective entitled “The Systems View of Man: Implications for Medicine, Science, and Ethics” has been published as early as in 1973 [1]. From a biological standpoint, systems biology is the large-scale dynamic study of functional and physical relationships between the molecules that make up life. This includes interactions within cells, between cells and between cells and their environments [2–5]. Systems biology aims to understand and describe complex biological systems and develop predictive models for physiological and pathological processes and apply them to control of disease states such as carcinogenesis. Four distinct aspects (system structure, system dynamics, system controls, and system design to introduce desired modifications) are considered in applying system biology approach to biological systems [6]. It is necessary to understand the functionality of interconnected complex biological networks in order to effectively devise appropriate cancer preventive measures and avoid any unwanted side effects. Numerous dynamic biological processes ranging from milliseconds (conformational changes) to minutes (post-translational protein changes) to hours and days (gene expression) and years (epigenetic control), maintain biological systems in certain quasi-equilibrium states. Application of engineering tools and concepts (e.g. networks, robustness, modularity, stochasticity, etc.) to biological studies is gaining increasing popularity and showing promise [6–8]. For example, integrative systems level approach has considerably increased the understanding of the EGFR signaling pathway, one of the most studied pathways [9]. However, it is also important to go beyond the cell and employ a more holistic approach in terms of the entire organism. For example, a role of cell-cell interaction/communication, locally and distally, has been implicated in carcinogenesis [10, 11] and is based on earlier made observations [12].

Biological systems in general and cancer in particular exhibit inherent resistance against internal and external perturbations, a characteristic termed robustness [9, 13–16]. Robustness differs from principles of stability and homeostasis in that it deals with maintaining system function as opposed to system states. This trait is ubiquitous in nature and largely due to extensive built-in redundancies (fail-safe mechanisms relying on alternative components or functionalities to maintain the system function), modularity (isolation of perturbation of one component on the whole system), decoupling (buffering of noise and fluctuations) and system controls via feedback loops (negative, positive, feed-forward) [6, 13]. However, it should be kept in mind that there is always a trade-off among robustness, fragility, resource demands and performance [6]. Kitano had proposed that cancer may be viewed as a breakdown of normal physiological robustness and change to pathological state that develops its own robustness in addition to using the host’s robustness [6, 15]. He had examined the theory of biological robustness in relation to cancer, inhibition of carcinogenesis, and drug design [6, 13] and proposed a need for cancer robustness theory motivated approach to cancer prevention and therapy [6].

One of the re-emergent theories of carcinogenesis involves cancer stem cells (see, http://dcp.cancer.gov/newsandevents/eventsarchive/20070514-15 and [17, 18].) Cancer stem cells represent a small subpopulation of cancer cells within a tumor and are characterized by their ability for self-renewal and pluri-potency. These cells are resistant to common intervention treatments and are thought to be responsible for cancer formation and growth, relapse and different stages of carcinogenesis. The microenvironment also plays an important bidirectional role [19].

Cancer is a dynamic multi-step, multi-mechanism disease involving complex interactive and redundant pathways, e.g. upregulation of survival pathways (e.g. bcl2, NFkB, AKT and receptor kinases) and genetic and epigenetic changes in relevant targets during cancer progression [20]. Consequently, it is important to understand the dynamic progression of cancer and apply appropriate preventive interventions accordingly. Heterogeneity, robustness, system dynamics and importance of different molecular targets changes during the carcinogenesis process greatly limit usefulness of a single “magic bullet” approach to intervention. There is a growing movement from a single target drug to multi-target drug paradigm [21] due to recognition that an alteration of a single target may be inadequate to produce desired biological effects. Instead, a partial modification of several targets may be more effective than a complete inhibition of a single target based on network models. Recently, an importance of targeting entire pathways has been strongly emphasized in [22, 23]. These authors conclude their work by the following notable statement [22]: “In addition to yielding insights into tumor pathogenesis, such studies provide the data required for personalized cancer medicine. Unlike certain forms of leukemia, in which tumorigenesis appears to be driven by a single, targetable oncogene, pancreatic cancers result from genetic alterations of a large number of genes that function through a relatively small number of pathways and processes. Our studies suggest that the best hope for therapeutic development may lie in the discovery of agents that target the physiologic effects of the altered pathways and processes rather than their individual gene components. Thus, rather than seeking agents that target specific mutated genes, agents that broadly target downstream mediators or key nodal points may be preferable. Pathways that could be targeted include those causing metabolic disturbances, neoangiogenesis, misexpression of cell surface proteins, alterations of the cell cycle, cytoskeletal abnormalities, and an impaired ability to repair genomic damage.” Vogelstein had further elaborated (www.bio-itworld.com/pb/2008/09/25/gbm-vogelstein.html): “By targeting the pathways, it’s possible new drugs could be effective against a much greater fraction of tumors. This is a very different perspective from what’s now operative in the drug development community”. In line with these ideas, it has been proposed in [21, 24, 25] that low affinity, multi-target drugs, representing weak links in cellular networks, may have a greater tendency to stabilize complex networks. Lack of effectiveness or presence of undesirable side effects have been ascribed to emphasis on drugs against a single target [15]. Single target interventions ignore redundancy, cross-talk, heterogeneity and pleiotropy. For example, selective inhibition of oncogenic AKT could have detrimental effects on glycogen metabolism which could be avoided by multi-component intervention downstream instead [3]. Another example of oncogenic pathway redundancy and crosstalk involves TGF-β [3]. Inhibition of TGF receptor would inhibit growth promoting SMAD2 and SMAD3, but would also activate oncogenic MAPK signaling. Inhibition of either SMAD2 or SMAD3 would likely lead to compensatory upregulation of its redundant counterpart. Many drugs have multiple targets and rational design of multi-target drugs will require much more temporo-spatial information about metabolic pathways, receptor signaling and signal transduction. While the reductionist approach has provided valuable information on individual molecular targets and their function, additional knowledge on spatial and temporal dynamic characteristics and complex interconnections in biological systems are needed for understanding and modulation of biological processes [8]. In fact, spatial and temporal dynamics of downstream signaling pathways may determine the specificity and nature of biological response [26]. Therefore, it is expected that application of systems biology to cancer prevention in terms of time dependent drug target selection should improve efficacy and decrease toxicity of preventive interventions. Drug combinations are common in antibacterial and cancer chemotherapy and traditional medicine. In fact, many drugs exhibit biological effects via multiple simultaneous activities at different targets [27]. Cancer prevention, like prevention of other complex diseases, would benefit from combination therapy based on dynamic systems biology approach as opposed to isolated, static view of the disease. There is a need to avoid reductionism and consider the entire biological system. It has been proposed that control of cellular dynamics may be more effective against cancer than that of its components. Therefore, a need for systems biology approach to improved understanding and control of disease progression and multicomponent intervention in network systems in general and cancer and cancer prevention in particular is obvious. Application of systems biology to complex biological systems is in its infancy but the need and rewards are great.

The most concise definition of the term “systems biology” is that systems biology is the theory of systems applied to biology. Theory of Systems, as a separate discipline with its own methodology, philosophy, mathematical instrumentation and fields of applications, has existed for almost a century. It is an interdisciplinary field of science which studies complex systems in nature and society such as an organism, organization, mechanism or informational network. Theory of Systems stems from the Bogdanov’s “Tectology” [28] and Bertalanffy’s “General System Theory” [29]. The General System Theory (GST) is widely regarded as an alternative view to that based on fundamental, often called *first*, principles of natural sciences. As such, the GST has introduced a number of new concepts and categories not reducible to those of physics, chemistry or biology. Among them are the concepts of complexity, adaptation, evolution, robustness, self-organization, catastrophes, chaos, criticality and numerous others. The GST stimulated development of a number of mathematical disciplines with central role of the concept of *network* and deep connections to graph theory and algebraic geometry.

By definition, a complex system is composed of interconnected parts that as a whole exhibits properties not obvious from the properties of the individual parts. Examples of complex systems include socio-economic structures, language, crowd psychology, termite colonies, biochemical networks, organizational culture, nervous system, social networks, cells and living things, internet, terrorist movements, energy infrastructure, traffic patterns, etc.

A number of prominent organizations in the U.S.A. and around the world are engaged in research and consulting pertaining to GST. Among them are the Santa Fe Institute (www.santafe.edu), RAND Corporation (www.rand.org), Center for the Study of Complex Systems (University of Michigan, www.cscs.umich.edu), Northwestern Institute on Complex Systems (www.northwestern.edu/nico), New England Complex Systems Institute (www.necsi.org), Department of Complexity Science and Engineering (University of Tokyo, www.k.u-tokyo.ac.jp/complex), Institute for Quantitative Social Science (Harvard University, www.iq.harvard.edu), and other.

The *Living Systems* theory [30] is an outgrowth of GST intended to formalize the concept of life. The discipline of *Systems Biology* is an aspect of the Living Systems theory which intends *to integrate* an ever growing body of knowledge about individual processes on all the levels of living systems using the conceptual frameworks of GST. There are (at least) three salient concepts crucial for understanding complex biological systems in addition to those existing in the GST. These are *emergence, robustness and modularity* [31]. The concept of *emergence* means that complex systems display properties that are not demonstrated by their individual parts and cannot be predicted even with full understanding of these parts alone. Comprehensive understanding of such emergent properties requires the system-level conceptualization and cannot be derived from the reductionist perspective focused on the system’s components. *Robustness* is an inherent property of all biological systems and consists in their ability to maintain functional stability in the presence of adverse influences imposed by the environment. Robustness is manifested through the feedback loops and other forms of self-control. A *module* is a functional unit possessing certain intrinsic properties regardless of its interactions with the external world. In biology, a module consists of the subunits that have strong mutual interactions and participate in common function. *Modularity* provides robustness to the system by confining the damage to a single part and preventing its spread throughout the system.

A wealth of information has been accumulated in the twentieth century regarding the individual cellular components and their functions. On top of the knowledge inherited from the past, an explosive influx of new data is currently emerging due to high-throughput technologies such as microarrays and protein mass-spectrometry. With such abundance of information, it becomes increasingly clear that complex biological functions cannot be generally attributed to individual molecules or molecular complexes such as DNA, mRNA or proteins. A key challenge for modern biology is to put forward an integrated approach capable of envisioning the system’s functionality from the properties of the individual parts of which it consists [8].

The scope of work in systems biology is enormous. Scientific journals with the key words “systems biology” in the title are numbered in dozens. A substantial fraction of the publications is devoted directly or indirectly to the systems biology of cancer. The discipline of *Mathematical Oncology* has emerged which attempts to integrate the gigantic and ever-growing body of knowledge on individual processes of tumor onset and proliferation with large-scale data mining, mathematical modeling and high-performance computing. Increasingly, cancer is seen as a “systems biology disease” [32] as it has become obvious that there is literally no hope to defeat cancer by mere isolating individual “targets” and inventing strategies for their modification. Development of a systemic view, however difficult, is in fact the only way to proceed. It is stated in [33]: “While the amount of gene expression data has explosively grown in recent years, an integrated theory of gene expression and regulatory network is not yet available. This divergence is the major bottleneck for making a progress in understanding biological systems.” This opinion is echoed by the NCI Strategic Plan, *Nation’s Investment in Cancer Research* (http://plan.cancer.gov). It states “integration of experimental biology with mathematical modeling will provide new insights in the biology and new approaches to the management of cancer.”

Against the backdrop of such monumental efforts in systems biology in general, and in the systems biology of cancer in particular, it seems almost surreal, if not regrettable, how small is still the role that applications of systems biology play in *cancer prevention*. It is noted in [34]: “Remarkably, despite the wealth of information, clinical oncologists and tumor biologists possess virtually no comprehensive theoretical model to serve as a framework for understanding, organizing and applying these data. Heeding lessons from the physical sciences, one might expect to find oncology aggressively, almost desperately, pursuing quantitative methods to consolidate its vast body of data and integrate the rapidly accumulating new information. In fact, quite the contrary situation exists.” It is not to say that there is lack of proposals in the literature to use various systemic approaches for identifying therapeutic and chemo-preventive targets (mostly based on experimentation with animal models and in-vitro human cell lines) [35]. Rather, that means that no integrated approach yet exists that would summarize existing consensus knowledge for application and decision making in the domain of cancer prevention. There are many reasons behind such a situation, not only purely scientific but also logistical, historical, cultural and socio-economic. It is much easier, however, to express frustration regarding the status quo rather than to propose a workable approach that would be both realistic in terms of available resources and capable of producing a noticeable impact in the near future. This paper is intended to provide a view on how to initiate a major effort to activate the role of systems biology in cancer prevention.

## A Glimpse of Mathematical Oncology

The language of the GST, in general, and the systems biology, in particular, is mathematics; complex systems require complex mathematics for their adequate description. A cursory look through the systems biology journals reveals an astounding array of mathematical disciplines which are in use for the description of complex biological systems. A long list of such disciplines is pioneered by the ordinary differential equations, partial differential equations and stochastic differential equations. Moving deeper, one can find Markov processes, cellular automata, graph theory, Boolean networks, chaotic dynamics, neural networks, and even such a highly abstract discipline of algebraic geometry. Historically, mathematical physics has been a cradle for the majority of powerful mathematical methods; these are in routine use in classical and quantum mechanics, astronomy, statistical physics, hydrodynamics, thermodynamics, optics, chemical kinetics, astrophysics, electrodynamics, wave dynamics, turbulence and many other areas. It therefore comes as no surprise that many of the approaches developed in physics have percolated into the theories of complex systems, systems biology including. However, it should be unequivocally stated that the objects being studied in biology are often much more complex than those in physics, and less amenable to formulation in terms of abstract models. It is therefore erroneous to assume that the methods of mathematical physics are overly difficult for use in biology. In fact, quite the contrary to this view, the methodologies offered by mathematical physics, despite being highly sophisticated and rigorous, are often *not complex enough* to reflect the biological realities. Traditionally, an experimental biologist is accustomed to collaborating with a statistician, and it is often the case in the biological community that applied statistics is thought to be the only mathematical discipline which is really necessary for understanding biological data. In reality, statistics is only a narrow slice in a vast body of mathematics. Statistical predictions are limited to the data at hand and are inherently incapable of comprehending global structure and dynamical patterns of behavior of big and complex systems. It is clear that all the empirical information pertaining to even the smallest fragment of a living organism such as, say, an individual act of gene expression or protein folding—let alone information about the interaction of tens of thousand genes and proteins—would probably be never possible to collect, no matter how much time, money and labor were poured into an experimental investigation. Therefore, the *unmodeled realities* of real organisms will ever be unattainable and unavailable for statistical evaluation. Only a conceptual dynamical model allows for playing the *what-if* games with big systems. Predictions in such systems cannot be reduced to the statistical ones; the hypotheses generated are those of global behavior, not of a short list of pre-selected predictors. Verifications of such predictions are not expressed in p-values. Rather, the most salient elements of the overall dynamics featuring an entire class of systems are the outcomes in such verification. Predictive force of dynamical models is not reduced to extrapolation to future of the data at hand. Rather, it allows for envisioning possible reaction of the system on the perturbations not actually observed in the experiment. It is not out of place to note that dynamical and statistical models are not the alternatives in data representation and analysis, but compliment each other. Having a conceptual dynamical model in place, a process called *data assimilation* becomes possible. In contrast to empirical model fitting, which is a fundamental building block in statistical analyses, the data assimilation assumes, schematically speaking, that the parameters in the *equations* describing the model are subject to fitting. The success of such a process is evaluated through similarity of the *solutions* to these equations with the phenomena experimentally observed [36, 37].

The field of computational oncology is flourishing. It is beyond the goals of this paper to give a systematic account of this field. The papers [38, 39] provide a good sense of the major accomplishments in this field but are far from being exhaustive either (the latter provides an extensive review of publications prior to 2003 and contains more than 300 references.) Very schematically, various aspects of mathematical oncology may be viewed as a sort of hierarchical structure. On top, one may find the works exploring the very concept of cancer as a genetic disease and general condition under which such an anomaly may occur. A notable example is [40] in which cancer is seen as a “robust intrinsic state of molecular-cellular network shaped by evolution.” According to this work, and also to [41–43], the genetic regulatory system, being multidimensional with strong nonlinear interactions, may posses a set of anomalous metastable states manifesting themselves as a genetic disease, although such states are not necessarily linked to any genetic damage or somatic mutation. The importance of this kind of works is that they challenge the somatic theory of cancer, the theory which dominates the mainstream of cancer research [44]. These works may be seen as meta-theories that attempt to comprehend cancer from the GST viewpoint. They basically convey the idea that any multidimensional highly nonlinear system—and genetic regulatory network is one of them—may have a dominant (or “normal” or “healthy”) dynamics, but also may be trapped in some secondary metastable states, which may be considered as “abnormal” and naturally associated with a disease. Since no actual damage exists in the system, spontaneous tunneling between different metastable states may be a purely stochastic process representing a natural way of life of the system. Hence, a remission would not be such a great miracle within this paradigm; it may be seen as moving the system back to the dominant, i.e. to normal state. There are experimental evidences in favor of such a view [45]. Obviously, if such a viewpoint proves to be realistic then the entire concept of cancer prevention may dramatically change.

On the next level down in the hierarchy of models in computational oncology, one may find numerous models of specific processes in specific organs. These works heavily rely on sheer computational power of modern computers and include as much empirical knowledge regarding these processes as possible. Schematically, they may be classified into two groups: the models for capturing known biology and the models for capturing unknown biology [46]. The first class of models provides the simulation frameworks for answering the questions similar to those regarding the effects of inhibiting particular targets against various cancer formations or other types of medical intervention. This kind of models is especially important as a practical support in *decision making*: it can help in understanding the mechanisms beyond the limits imposed by available observations, outline the priorities and reveal weaknesses in existing paradigms. The second type of models is more suitable in *research settings* and fulfills the goal of incorporating the knowledge inferred from observational data into the existing theoretical models.

A large number of works in computational oncology are devoted to various aspects of cancer cell proliferation and tumor growth. The variety of theoretical approaches is astounding: “from diffusion models of avascular tumours to multiphase models of vascular tumours, from travelling wave analysis of tumour invasion to models of cell migration by chemotaxis in multicell spheroids, from multi-species fluid models to single phase viscoelastic models, from stochastic models of metastases formation to multiphase models of necrosis formation” [38]. An important aspect of these works is modeling angiogenesis as a key element in the development of invasive cancers [39].

Another large class of mathematical models in computational oncology is dealing with the dynamics of gene-to-gene and gene-to-protein interactions within intra-cellular regulatory networks. An extensive review is given in [47]. These models reveal the roles of genes and proteins in cellular processes and formation of the nodes for information exchange between signaling pathways. The gene expression profiles of cancer cells provided by microarrays are often used as empirical basis for reconstructing genetic regulatory networks. The models describing individual processes in mathematical oncology may serve as prototypes of the modules for future integration into a comprehensive all-encompassing computational model. Integration of the modular elements, both theoretical and empirical, into a single system may become a valuable resource for elucidating human diseases [48, 49].

Genetic alterations such as point mutations, chromosomal aberrations and DNA modifications accumulate during the lifetime of an organism. Each of these modifications of the molecular structure, either spontaneous or environmental, contribute to the DNA damage. Generally, the DNA replication in normal human cells is an extremely accurate process with probability of error less than 10^−9^ per nucleotide. Propagation of DNA damage through the subsequent generations of cells is an essentially stochastic process. Its modeling helps to envision loss of fidelity of replication due to the initial DNA damage. A number of sophisticated mathematical models have been developed to elucidate this issue (see [50] and an extensive bibliography therein.).

A focal point in cancer-related functional genomics is to understand how genetic or epigenetic perturbations to intra-cellular dynamics may lead to a disease. It should be noted, however, that there is no such thing as a time-invariable portrait of the cell, whether normal or cancerous; random temporal and spatial variations are ubiquitous patterns in gene expression. Therefore, the very notion of genetic perturbation requires for careful substantiation. Randomness and stochasticity are persistent topics in the dynamics of genetic regulatory systems. In particular, the phenomenon of “burstiness”, i.e., large sporadic variations in protein and mRNA concentrations, has received much attention in the literature [51–53]. Practical importance of this all-pervading phenomenon is twofold. First, such sporadic variation can be easily mistaken for erratic behavior of the cell and misinterpreted as a genetic disease. Second, intrinsic stochasticity and temporal variability impose certain limitations in interpretation of microarray experiments and their usage for prediction of cancer outcomes, especially in clinical settings (see the works [54–57] by one of the authors and references therein.) A comprehensive review of stochasticity in transcriptional regulation is also given in [58].

## Big Model: What is Involved?

As seen from the brief review presented in the previous section, the number of important processes associated with cancer onset and proliferation may be counted in hundreds, and the number of mathematical and computational methodologies to model these processes may be counted in thousands. Such an abundance of the models in circulation, however, does not make the life of a practitioner and a decision-maker in the field of cancer prevention any easier. To date no attempts have been made to design, or even envision, a comprehensive meta-model with the specific goal to be used in cancer prevention.

When attempting to outline a general structure and possible directions of development of such a model, several considerations come to mind. First, it should be mentioned that any single work in computational oncology is intended to elucidate certain processes of cancer onset and proliferation, and therefore potentially may help, directly or indirectly, to the field of cancer prevention. The problem is that these individual contributions, however important, do not translate directly into any therapeutic intervention or decision making in the domain of practical cancer prevention. One researcher, or a small group of researchers, working in the field of cancer systems biology have every right to claim that their efforts constitute, at least implicitly, a contribution to the cancer prevention. But a medical practitioner, policy maker, or program manager cannot be automatically assumed to be an expert in all the mathematical methods and biological interpretations available in the literature, and as a result it is often the case that they have no easy ways to evaluate their applicability to practical problems. Only when and if the individual models are integrated into a comprehensive system equipped with a user-friendly interface, can they become valuable assets in the science of cancer prevention.

Second, a realistic systems biology approach in cancer prevention is not supposed to serve by itself the purposes of scientific experimentation or hypotheses generation. Whenever possible, it should be based on the data which are considered established with some degree of consensus in the scientific community. In this sense, the goals of application of systems biology to cancer prevention are distinctly different from other, purely scientific, areas. The *hot topics* with much arguments and controversies around them would be a poor basis for practical solutions until they cool down to the point of crystallization into a solid and comparatively coherent scientific view. Although an ultimate truth could probably never be achieved, the criterion of being relatively well substantiated seems to be a reasonable filter for inclusion into the integrated meta-model in cancer prevention.

Third, cancer is a highly heterogeneous disease with many different spatial and temporal scales. On each of these scales, different conceptual, mathematical and computational tools are required to depict the corresponding processes, and it would be nearly impossible to create a “theory of everything” in cancer and implement it in a single model. Such a situation is quite typical in the world of big multi-scale models, and the only solution invented so far consists of constructing a modular (or compartmentalized) hierarchical system of sub-models working concurrently and providing all the necessary information to the higher hierarchical level. Numerous examples of this kind exist in many domains other than systems biology. In a very general sense, the systems biology for cancer prevention has a lot to borrow from the expertise accumulated in other sciences.

## Less is More in the Systems Biology for Cancer Prevention: Dynamics by Rules

Complexity of the biological processes associated with cancer onset and proliferation does not leave any hope for the success of any simple-minded reductionist approach in cancer prevention; invasion of the system-wide computerized methods seems inevitable. Developing a *comprehensive* mathematical model serving the purposes of cancer prevention may seem to be a pure fantasy today. Nevertheless, given the historical precedents, exponential growth of knowledge, fast penetration of mathematical culture into biology, and widespread availability of powerful computers, it is quite possible that within a decade or so any cancer prevention practitioner or clinical oncologist will have a comprehensive computational assistant running on his or her laptop. At this point, the question is not how to create such a big model from the scratch but how to *begin* the process of its creation with this ultimate goal in mind.

Although “thinking big” is useful, the first practical steps cannot be anything but small compared to the distance to cover. It is noted in the paper [59] with a telling title “Less is more in modeling large genetic networks” that “a central question is what the right level of description is when constructing quantitative models of large or even system-wide model of genetic networks.” A similar question may be posed with respect to any big model in the systems biology: how much detail is to be included into the model? Obviously, too much detail may be prohibitively costly in terms of time and labor for collecting the observational data and developing the mathematical model. On the other hand, an excessively crude model may deprive a system of its essential individual traits, thus reducing the model to an abstract formalized exercise. Sometimes, when a system has a certain degree of internal homogeneity, it is possible to apply a *coarse-grained* approach in which the functionality of individual elements is replaced by comparatively crude surrogate representation. Many such approaches are known in the dynamics of genetic regulatory networks; a useful review and extensive bibliography may be found in [60]. The coarse-grained approach is not, however, universally applicable, especially when the functionality of individual subsystems require drastically different mathematical tools for their description. Also, a general rule of thumb in developing big computational systems is that mathematical concepts appropriate at a certain level in the hierarchy of models are not generally applicable at the levels up or down in this hierarchy.

A viable approach to modeling big systems in biology has been recently proposed in [61]. This approach is a variant of the so-called *expert systems* (ES) and is well known in many sciences and applications [62]. An ES is an artificial intelligence framework which attempts to reproduce and automate the performance of a human expert or a group of them. The theory of expert systems has many links to the GST, applied mathematics, operations research and management science. In an ES, an expert formulates his/her knowledge in the form of verbal rules, generally avoiding mathematical notation. In formal terms, these rules consist of a series of definitions and atomic statements (i.e. those not reducible to a collection of simpler ones) and may be manipulated in accordance with the laws of formal logic. As such, they constitute the basis for computer algorithms which may be programmed, debugged, checked for self-consistency, augmented, coupled with other algorithms and included in a bigger system. An outcome of a rule-based algorithm is also a certain rule; therefore, the entire rule-based expert system may be replaced, in principle, by the module which generates the rules much more complex then the atomic expert rules. Thus, the set of such modular algorithms may be then assembled into a bigger system, producing a problem-solving tool of unlimited complexity.

There are multiple benefits of using the rule-based ES. The first advantage is that it provides a well-tested framework for formulating imprecise knowledge. It is not out of place to note that being *quantitative* is not a synonym to being *precise*. Quantitative models require numerical parameterization and explicit formulation of the functional form of equations. In reality, quantitative parameters are frequently known only within large margins of errors, if known at all. As to the selection of the equations’ functional forms, a mathematician/modeler is usually bound by the requirements of simplicity, solvability and computability. Subjective intuitive judgments are implicit participants in the development of any such system. Therefore, the quantitative representations often may not be more precise than the rule-based ones.

Second, a remarkable aspect of using the rule-based ES is that *in principle* they are capable of representing precise knowledge with any pre-specified accuracy, provided such knowledge is available. The way it may be done is through using the so-called fuzzy logic (FL). As known from the theory, FL models are capable of representing complex systems to high degrees of accuracy through a series of successive FL refinements and augmentations. The standard additive model (SAM), a common formulation of a FL system, is known to be a universal approximator, that is, to be in principle capable of approximating any nonlinear function as precisely as desired. In addition, FL logic models are naturally robust with respect to noise and variation in the system’s parameters, thus allowing for computation of the system’s dynamics with imprecise variables [61].

The third advantage is that formulation in the form of rules generally does not require an expert in the subject matter field to be also an expert in mathematics and/or computer science. Nevertheless, the formulations he or she provides constitute a valid basis for the algorithm development, programming and simulation experiments. Moreover, the ES statements are capable of depicting certain elements of knowledge when precise representation is unavailable. As an example, let us consider the following statement: “p53 protein is a transcription factor that functions as a tumor suppressor.” True or not, this statement spans over several scales of biological events, from the molecular level of events like gene expression, to the cellular level of events like cell cycle and apoptosis, to the tissue level of events like tumor growth and proliferation. Creation of a quantitative mathematical model for such a multi-scale process would be a daunting task by itself; nevertheless, the above-mentioned qualitative statement regarding the p53-protein may serve as a valid piece of information in a rule-based ES/FL. It is also worth mentioning that the commonly used graphical representations of metabolic pathways is nothing else than a set of fuzzy statements loosely connected into a bigger integrated scheme, thus being a variant of an ES/FL. Finally, big models based on *precise* equations usually produce a wealth of redundant information which is difficult to comprehend unless it is summarized into a set of concise, and inevitably fuzzy, rules.

Therefore, we come to the conclusion that ES/FL system is not a *poor man’s systems biology*; quite the contrary, it is a natural intermediate step towards an all-encompassing mathematical model. Several real-life examples of ES/FL models for complex biological processes are given in [61]. Java-based software framework of the ES/FL system specifically oriented towards biological applications is given in [63]. Fuzzy logic is a well developed mathematical discipline with numerous applications in science, engineering, medicine, systems control and other areas requiring use of artificial intelligence. Theoretical foundations of qualitative reasoning and simulation are described in-depth in [64] and implemented in the software package QSIM freely available from the University of Texas [65]. Application of ES/FL in the systems biology for cancer preventions would not be like testing the unknown rough waters; rather it would be an adaptation of a well tested tool to a new area of applications.

## World Dynamics Approach

Another popular methodology in constructing big compartmentalized multi-scale models is known as the *world dynamics* approach (WD). The term stems from the famous work *The Limits to Growth* [66] sponsored by the *Club of Rome* in early seventies. In this work, an attempt has been made to create a large-scale model of global industrial development in its competition with growth of the world population, exhaustion of natural resources and deterioration of environment. From the technical standpoint, the WD approach to modeling consists in combination of differential equations for the well established dynamical processes with empirical relations for the processes with unknown dynamics. Regardless of the success or failure of the WD in the domain it was originally designed for, it has become a valuable tool in large scale computational modeling. At the time of its introduction, the WD model was considered as very big and was able to be run only on the most powerful computers of that time. Nowadays, however, such a model would easily run on a modest quality laptop. Much more powerful WD models are currently available, and many of those are also suitable for working on personal computers. In particular, the ModelMaker software developed by ModelKinetix (see www.ModelKinetix.com) provides a computational environment for in-depth modeling in chemistry, environmental science, physiology, sociology, epidemiology, pharmacokinetics, economics, business management, ecology and mathematics. The WD approach is an appropriate basis for quantitative solutions of systems biology problems as well.

## Big Questions Regarding Big Models

### Computational stability

As mentioned above, behavior of a complex system consisting of interconnecting simple parts cannot be readily envisioned from the individual properties of these parts. The same may be said about big modular multi-scale computational models. There are a number of fundamental questions pertaining to general patterns of behavior of complex hierarchical systems, and perhaps the most important among them is the question of stability. There are several different aspects of stability and all of them are important in practical applications. First, one needs to consider the stability with respect to variations of parameters determining the analytical and/or logical structure of the model. An overall pattern of the model’s behavior may be largely independent of some of these parameters, whereas others may be critically important in the sense that their slight modification may cause a complete change in the model’s dynamics. Such a phenomenon is usually called bifurcation (or “branching”). Obviously, the parameters which found to be critical require more attention in terms of their accuracy and efforts to understand the origin of such criticality. This kind of sensitivity analysis may be seen as an important practical application of a computational model.

In the time-course dynamics, an important issue is the sensitivity with respect to variations of initial conditions. A viable computational system for simulating real life processes (such as pharmacokinetics in drug discovery, for instance) should not be too much dependent on initial conditions. Otherwise, all the predictions resulting from the simulation will be strongly dependent on the individual history of the simulated processes thus loosing their generality and practical value. There are a number of powerful mathematical tools for studying stability with respect to initial conditions with the *Lyapunov exponents* being in the center of all the relevant theories [67].

The question of sensitivity to variations of initial conditions is closely related to a more general question of overall dynamical stability. This question leads to the very depth of the dynamical systems’ behavior. Generally a big nonlinear system of equations may have a set of equilibrium (a.k.a. *fixed*) points and these may be stable or unstable. The importance of these concepts for multidimensional multi-scale modeling follows from the fact that only a stable system may have an asymptotic solution, and this solution is largely independent of the initial conditions (within certain *basins of attraction.*) In modeling, if the system is unstable then an apparent convergence of the solution to a certain limit may be a pure computational artifact having nothing to do with reality of the system-to-be-modeled. The question of dynamical stability in constructing the large computational models is just another aspect of the famous question posed by R. May: “Will a big and complex system be stable?” [68]. A general answer is that the probability of a system being stable is miniscule unless special efforts are undertaken *to design it to be stable*. Therefore, the question of stability should be of primary importance in developing a big computational model in the systems biology for cancer prevention. It is worth noting that many of existing software packages, although claiming to be universally applicable to modeling the biological networks, leave the question of stability largely unaddressed. An in-depth discussion of dynamical stability with application to biochemical networks has been recently published by one of the authors [69].

### Fast and slow variables, stochasticity

A big multidimensional computational model is necessary multi-spectral, i.e. includes the modules for the processes with drastically different characteristic time scales. A review pertaining to this issue in the context of modeling cancer is given in [70]. For example, mRNA production is the process with characteristic times in minutes, cell cycle takes from hours to days, and tumor growth is a process with time scales from months to years. In computational models, it is neither practical nor technically possible to maintain the same time scale for the entire system; some kind of reduction in the state variables is unavoidable. A number of techniques have been developed in computational mathematics to solve this problem with two of them having gained a wide popularity: the first one is known as the *principle of enslaving* [71], and the second as the *elimination of fast variables* [72]. In the former approach (playing a prominent role in the mathematical models of self-organization), the *slow* processes are considered to be *frozen* at any moment of *fast* time, thus providing a constant background for the fast processes. After resolving all the equations for fast processes, their summaries (e.g. averages) are being fed into the model for slow variables. Thus, fast processes become parameterized by slow evolving background. In the latter approach (playing a fundamental role in the stochastic dynamics of nonlinear systems) the *fast* variables are considered to be *chaotic* in the *slow* time and replaced by an appropriately constructed stochastic process. Slow time differential equations are replaced by the stochastic differential equations with the diffusion tensor obtained from the fast scale. There are innumerable variants of these two key ideas in computational science and they are entirely relevant in the models for systems biology in cancer prevention as well.

## Where to Start?

A starting point for any further development is a mere recognition of the fact that the systems biology models specifically designed to be used in cancer prevention are currently nonexistent. Even such a simple action as start moving somewhere requires strategic vision, organizational efforts, resources, motivated people and time. Although ultimately the model may be very big, the first steps are necessarily small. These small steps, however, should be in the direction of *integration* rather than towards further elaboration of individual processes and their in-depth mathematical modeling. In fact, the mass of the knowledge currently available is so monstrously huge that it may have already passed the point of being manageable. There is a serious risk of completely losing this knowledge for any practical purpose unless decisive steps towards integration are undertaken.

Whatever the direction for further steps is selected, certain initial actions seem unavoidable and at the same time economical. They consist in accumulation of the verbal expert summaries in any well established domain of preventive oncology. Whenever possible these summaries should follow common rules and common terminology. Scientific organizations with a modular structure, where each research group is focused on certain types of organs/cancers, are especially well suited for these purposes. In a sense, their modularity may mirror the modularity of a future compartmentalized mathematical model. Importantly, at this stage of development no serious involvement of mathematicians and/or computer scientists is required; although coordination and unification would be highly desirable. All the summaries may be stored in databases containing the sequences of subject matter statements. There are special algorithmic languages capable of processing these sequences in an automatic manner, with PROLOG being the best known example. From this point on, there are many ways to proceed towards quantitative representation of the processes of interest. In particular, an elegant way of creating a semi-quantitative model from purely qualitative rule-based information is the technique known as Qualitative Differential Equations (QDE) [73]. In this approach, fuzzy statements from ES/FL are replaced by their quantitative analogs taken from the pool of pre-defined functional relations. For example, the statement “Y grows with X” may be replaced by the linear function, statement “F periodic with time” may be replaced by the sinusoidal function, and so on. This process is well formalized, may be performed in a more or less automatic fashion, and may result in a fairly complex quantitative model. On the other hand, already existing *genuinely quantitative* models analyzing the details of corresponding fuzzy statements may be included into the system *as is* or after appropriate *fuzzification*. The latter means, for example, that a very complex behavior obtained from the solution of differential equations may be summarized as a combination of fuzzy statements like “proportional”, “growing fast”, “periodic”, etc, thus bringing complex mathematical language closer down the earth of informal subject matter thinking. After all the modules comprising the system are described and tested in a rule-based or semi-quantitative manner, the full power of mathematical and computational methods may be applied selectively to those modules which are found to be really critical and do require to be analyzed in fine details.

## Conclusion

It is becoming increasingly recognized in scientific community that a systems biology approach should prove invaluable and even necessary to understand, simulate, predict and control complex biological processes such as carcinogenesis and to develop effective strategies in cancer prevention. We have outlined the status quo and possible ways of development of a computerized model specifically oriented towards application in cancer prevention. In particular, it has been proposed that three approaches, namely the rule-based fuzzy logic *expert systems*, the *world dynamics* type of models, and the *qualitative differential equations*, taken separately or in combination, would constitute an appropriate basis for initial steps in development of a large computational and informational framework with focus on cancer prevention. It is not our intention in this paper to claim that these ideas are the only ways to proceed. Rather, our goal is to initiate a discussion in the cancer prevention community of the pros and cons of various approaches and to start a major movement in this direction. At this point, all options are on the table, and time is of the essence.
